# Quantitative evaluation of sweet-responsive type II cells in mouse taste buds via biocytin uptake

**DOI:** 10.1007/s00418-025-02442-w

**Published:** 2025-12-06

**Authors:** Katsuhiro Kaetsu, Hiroyuki Nakagawa, Takashi Yamasaki, Yoshitaka Ohtubo

**Affiliations:** 1 Graduate School of Life Science and Systems Engineering, Kyushu Institute of Technology, Hibikino 2-4, Kitakyushu, 808-0196 Japan; 2 Department of Chemical and Biological Engineering, National Institute of Technology (KOSEN), Sasebo College, Okishin-cho 1-1, Sasebo, 857-1193 Japan; 3 Division of Biology, Faculty of Science, Fukuoka University, Nanakuma 8-19-1, Fukuoka, 814-0180 Japan

**Keywords:** Biocytin uptake, Taste stimuli, Membrane depolarization, Signal transduction, Mouse

## Abstract

Sweet taste is mediated by type II taste bud cells (TBCs), which express the heterodimeric taste receptor composed of type 1 members 2 and 3, a G protein-coupled receptor. Activating this receptor triggers phospholipase Cβ2 (PLCβ2)-dependent signaling, depolarizes cell membrane, and leads to ATP release via calcium homeostasis modulator 1 and 3 channels. However, the number of sweet-responsive cells within individual fungiform taste buds remains poorly understood. To quantify the number of sweet-responsive TBCs, we developed a novel method using biocytin uptake as an indicator of membrane depolarization. The apical side of peeled mouse lingual epithelia was stimulated with 1 M sucrose or 30 mM saccharin, while biocytin was applied to the basolateral side. Sweet stimulation significantly increased the number of biocytin-labeled cells compared to deionized-water controls. Biocytin labeling was observed primarily in PLCβ2-positive type II cells, with additional labeling in PLCβ2 and synaptosomal-associated protein 25-negative cells, suggesting the involvement of type II and, likely, type I cells. On average, 11% of type II cells per taste bud were sweet-responsive; however, this proportion varied substantially across individual taste buds. These results indicate that sweet-responsive cells form a subset of type II cells and are distributed heterogeneously among fungiform taste buds. Such heterogeneity may reflect divergent tuning properties and contribute to robust sweet taste perception. Given the short lifespan and continuous turnover of TBCs, asynchronous renewal of sweet-responsive cells across taste buds may help maintain sweet sensitivity by ensuring that some sweet-sensitive cells are consistently present.

## Introduction

A taste bud consists of 10–100 taste bud cells (TBCs), which are classified into four types (types I–IV) based on their morphology and functions (Chaudhari and Roper 2010; Murray 1973; Roper 2013). Among these, type II TBCs express G protein-coupled taste receptors responsible for detecting sweet, bitter, and umami substances (Adler et al. 2000; Nelson et al. 2002, 2001). When activated, type II cells release ATP via paracrine signaling (Finger et al. 2005), where it functions as a transmitter for gustatory nerves. Although type II cells constitute approximately 25% of the cell population in individual taste buds in mice (Ogata and Ohtubo 2020; Ohtubo and Yoshii 2011), the proportion specifically responsive to sweet stimuli remains unknown.

Sweet taste perception primarily involves heterodimeric receptors formed by taste receptor type 1 member 2 (T1R2) and taste receptor type 1 member 3 (T1R3) subunits (Nelson et al. 2001). Activation of the T1R2/T1R3 receptors triggers a signaling cascade involving phospholipase Cβ2 (PLCβ2) and inositol 1,4,5-triphosphate (IP_3_), leading to changes in the intracellular Ca^2+^ concentration and subsequent membrane depolarization. These events ultimately lead to ATP release through calcium homeostasis modulator 1 and 3 (CALHM1/3) channels (Kashio et al. 2019; Taruno et al. 2013). A recent study demonstrated that, in response to saccharin stimulation, taste receptor cells generate oscillatory receptor potentials accompanied by action potentials in a concentration-dependent manner (Nakao et al. 2022). Such oscillatory membrane depolarizations are thought to facilitate the opening of large-pore, ATP-permeable channels, allowing the entry of extracellular molecules such as biocytin into cells. Indeed, previous studies have demonstrated that membrane depolarization induced by the application of a high-K solution to the basolateral membrane promoted biocytin uptake into TBCs (Iwamoto et al. 2020; Takeuchi et al. 2011).

In this study, we quantified the number of sweet-responsive cells within individual taste buds by selectively applying sweet substances to the apical membranes of TBCs and using biocytin uptake as an indicator of cellular activation. The results revealed that biocytin uptake occurred primarily in type II cells, with an average of approximately 11% of type II cells per taste bud showing sweet responsiveness. Interestingly, substantial variation in the number of sweet-responsive cells was observed across individual taste buds. We discuss the implications of this variability in sweet responsiveness among taste buds.

## Materials and methods

### Preparation of peeled lingual epithelium

Peeled lingual epithelium was prepared as described previously (Mori et al. 2016; Ohtubo 2021; Ohtubo et al. 2001). In brief, following anesthetization with CO_2_, 5–8-week-old male ddY strain mice were killed by decapitation, and the tongues were removed. An elastase solution was then hypodermically injected into the tongue, and the tongue was incubated at 25 °C for 3–4 min. Then the epithelium containing fungiform papillae was peeled off using forceps under a stereomicroscope. The peeled epithelia were mounted on a recording platform, with the basolateral membrane side of the TBCs facing upward. Overall, tongues from 21 mice were used to prepare peeled lingual epithelial samples for response measurements.

### Biocytin uptake

The biocytin uptake experiments were performed by controlling the solutions applied to the apical and basolateral sides of the membrane. First, the apical membranes of the TBCs were immersed in deionized water for 1 min, while the basolateral membranes were immersed in physiological saline. This adaptation with deionized water was repeated twice. Next, the basolateral membranes were immersed in physiological saline containing 2 mg/mL biocytin, while the apical membranes were immersed in the taste substances for 5 or 10 min. Finally, the apical and basolateral solutions were replaced with fresh deionized water and physiological saline, respectively, and held for 1 min; this process was repeated twice. For the high-K stimulation, the basolateral membranes were immersed in 150 mM KCl solution containing biocytin for 5 min, while the apical membranes were immersed in deionized water. Subsequently, the basolateral membranes were rinsed twice in physiological saline for 1 min each time. Experiments were conducted at either 25 °C or 38 °C using prewarmed solutions.

### Visualization of biocytin-labeled TBCs and identification of cell types

To identify the cell type of the biocytin-labeled TBCs, we stained the peeled epithelium with cell-type-specific antibodies, as described previously (Takeuchi et al. 2021, 2011). In brief, the peeled epithelia containing biocytin-labeled TBCs were fixed with 4% paraformaldehyde in phosphate-buffered saline (PBS) for 24–48 h at 4 °C and then pre-treated with blocking solution containing 3% normal donkey serum, 0.3% Triton X, and 1% bovine serum albumin in PBS for 3 h at 25 °C. Then, immunostaining was performed using primary antibodies dissolved in blocking solution for 24–48 h at 4 °C. After washing in PBS, the epithelia were incubated with Alexa Fluor-conjugated secondary antibodies and Alexa Fluor 633-conjugated streptavidin (1:100, S21375; Molecular Probes, CA, USA) for 24–48 h at 4 °C.

In this experiment, we selected the cell-type marker PLCβ2 for type II cells and synaptosomal-associated protein 25 (SNAP-25) for type III cells, in accordance with previous reports (Ogata and Ohtubo 2020; Ohtubo and Yoshii 2011). Anti-PLCβ2 rabbit polyclonal antibody (1:100, sc-206; Santa Cruz Biotechnology, TX, USA) and anti-SNAP-25 mouse polyclonal antibody (1:500, S5187; Sigma-Aldrich, MO, USA) were used as the primary antibodies, while Alexa Fluor 488-conjugated donkey anti-rabbit IgG (1:400, A21206; Molecular Probes) and Alexa Fluor 555-conjugated donkey anti-mouse IgG (1:400, A31570; Molecular Probes) or Alexa Fluor 546-conjugated donkey anti-mouse IgG (1:400, A10036; Molecular Probes) were used as the secondary antibodies.

**Fig. 1:**
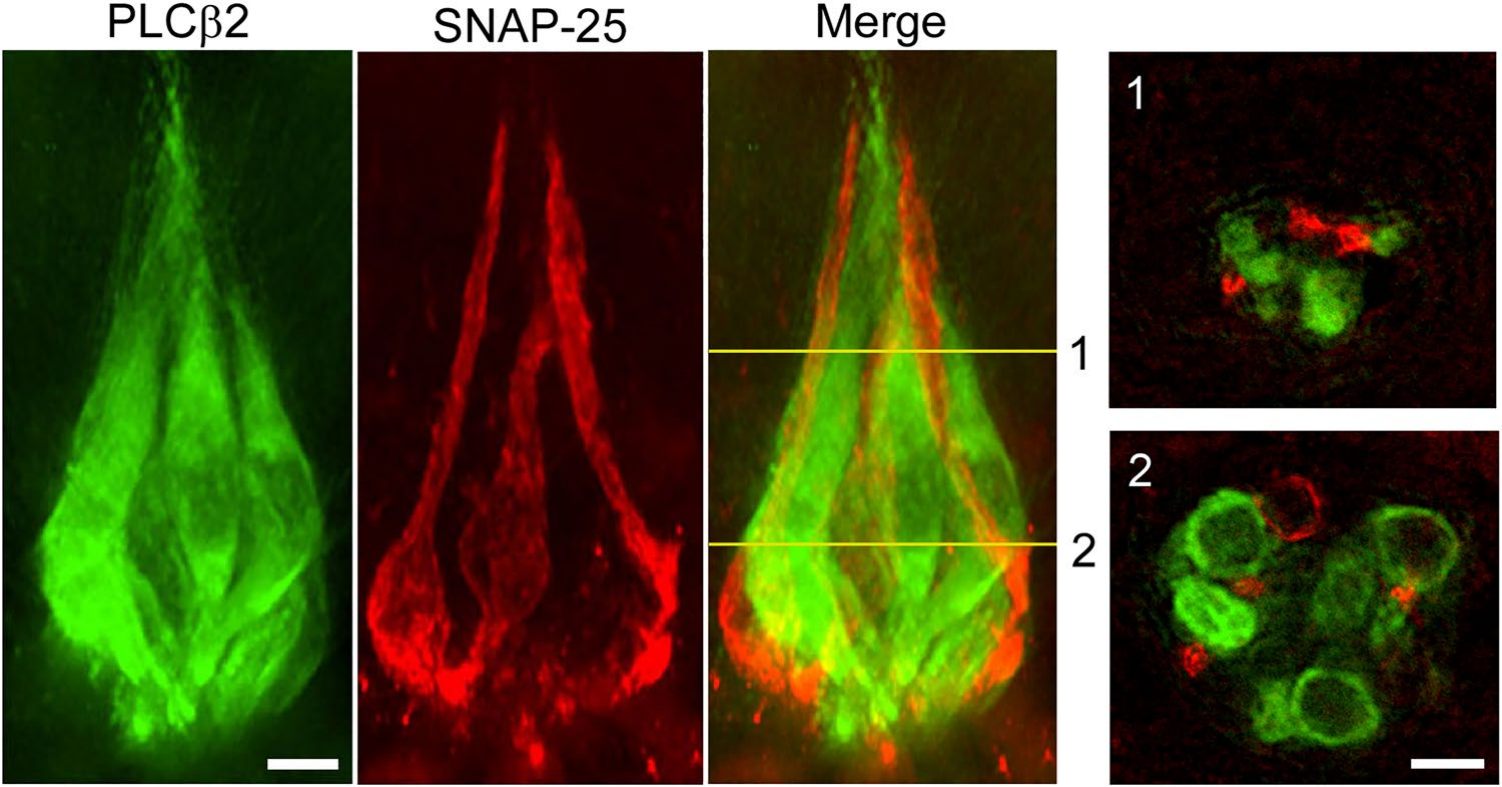
Three-dimensional reconstruction of a fungiform taste bud. The apical surface, containing the taste pore, faces upward, whereas the basal side faces downward. The rightmost images labeled “1” and “2” are orthogonal cross sections corresponding to the positions indicated by lines 1 and 2 in the merged image. PLCβ2 (a type II cell marker) immunoreactivity is shown in green; SNAP-25 (a type III cell marker) immunoreactivity is shown in red. Scale bars 5 µm

**Fig. 2:**
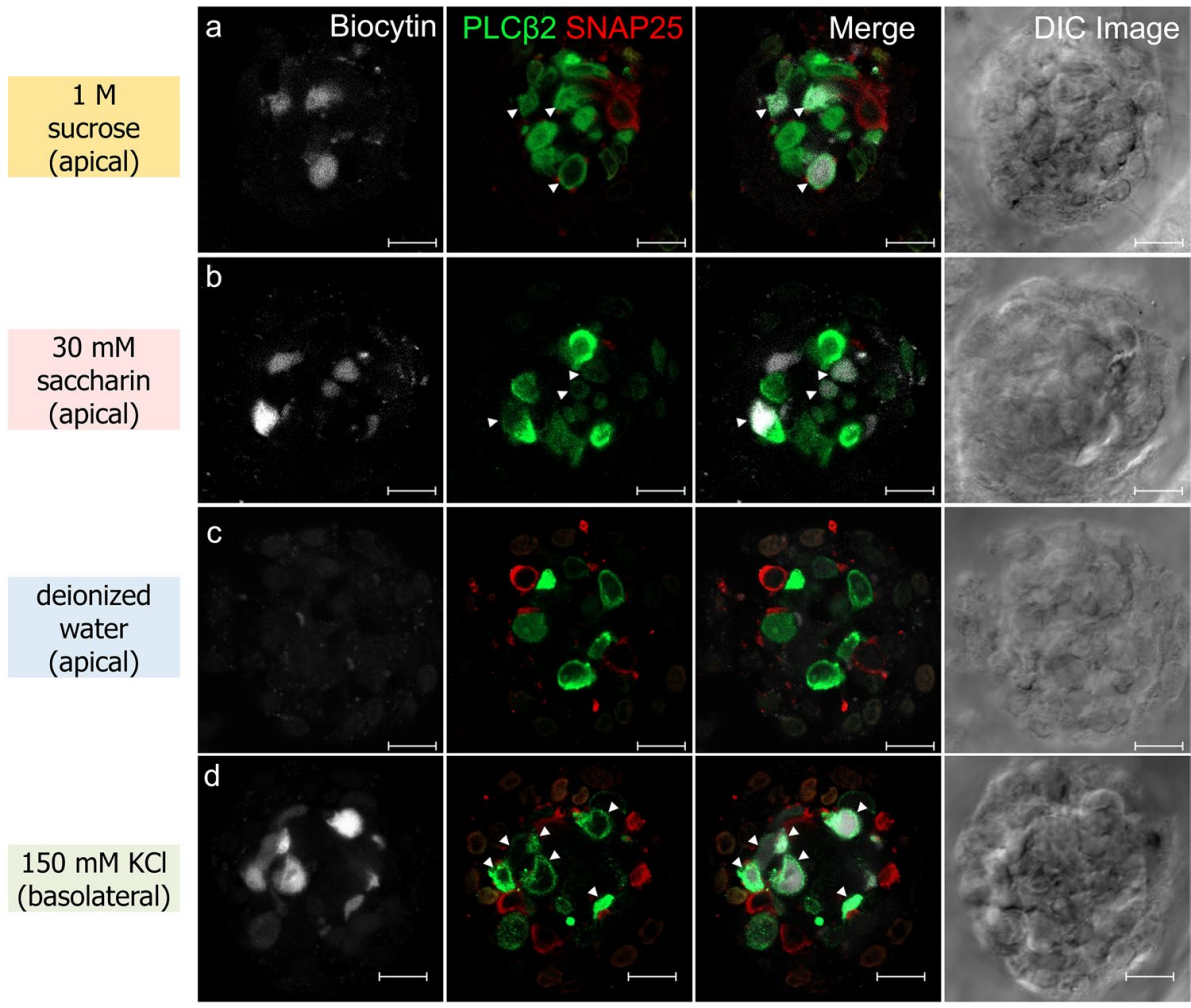
Exposure of taste bud cells (TBCs) to sucrose or saccharin solution applied exclusively to the apical membrane induces biocytin uptake. TBCs were labeled with biocytin in response to **a** 1 M sucrose, **b** 30 mM saccharin, and **c** deionized water applied to the apical membrane for 5 min. **d** Biocytin uptake also occurred upon exposure to high-K solution (150 mM KCl) applied to the basolateral membrane. After biocytin labeling, the same taste buds were subjected to immunohistochemical staining with antibodies against PLCβ2 and SNAP25. Columns from left to right show biocytin labeling (white), PLCβ2 immunoreactivity (green) and SNAP25 immunoreactivity (red), merged images, and the corresponding differential interference contrast (DIC) image. Arrowheads indicate biocytin-labeled type II cells. Scale bars 10 µm

The immunostained epithelial preparations were viewed under confocal microscopy (LSM980; Carl Zeiss AG, Oberkochen, Germany) as described previously (Iwamoto et al. 2020; Ogata and Ohtubo 2020). Confocal images were obtained over the entire length of taste buds and were averaged from two or three images for each focal plane, with sequential acquisition in 1.5-µm steps at the optimal wavelength for the respective fluorescent dye. Averaged confocal images were analyzed using ZEN (ver. 3.4; Carl Zeiss Microscopy GmbH) and Fiji, an image processing package based on ImageJ (ver. 1.54 g; Wayne Rasband and contributors National Institutes of Health, USA). The number of immunoreactive cell somata was counted for each immunoreactive cell type in a single taste bud, as previously described (Ogata and Ohtubo 2020; Ohtubo and Yoshii 2011). In brief, the somata of TBCs were identified as immunohistostained rings (Figs. 1 and 2). A region was determined to be immunoreactive when the fluorescence intensity was greater than twice the background level. We identified an immunohistostained ring as a soma when it appeared on at least three continuous images (4.5 μm). The number of somata was counted throughout the individual taste buds.

Three-dimensional images of fungiform taste buds labeled with cell-type markers were obtained and analyzed using a super-resolution structured illumination microscope (N-SIM; Nikon Corporation, Tokyo, Japan). Z-stacks spanning the entire apico-basal axis were sequentially captured at 0.16-µm axial intervals with excitation and emission settings specific to each fluorescent dye. Three-dimensional renderings were produced and presented as maximum-intensity projections.

### Solutions

All solutions were prepared using deionized water and analytical grade reagents, which were purchased from Wako Pure Chemical Industries (Osaka, Japan), unless otherwise stated. The physiological saline solution contained 150 mM NaCl, 5 mM KCl, 2 mM CaCl_2_, 0.5 mM MgCl_2_, 10 mM glucose, and 5 mM HEPES–NaOH (pH 7.4; Sigma-Aldrich, St. Louis, MO, USA). The high-K solution contained 5 mM NaCl, 150 mM KCl, 2 mM CaCl_2_, 0.5 mM MgCl_2_, 10 mM glucose, and 5 mM HEPES–KOH (pH 7.4). The elastase solution consisted of 1 mg/mL elastase (high purity from porcine pancreas) dissolved in physiological saline. The PBS contained 137 mM NaCl, 8.1 mM Na_2_HPO_4_, 2.68 mM KCl, and 1.47 mM KH_2_PO_4_.

## Results and discussion

**Fig. 3:**
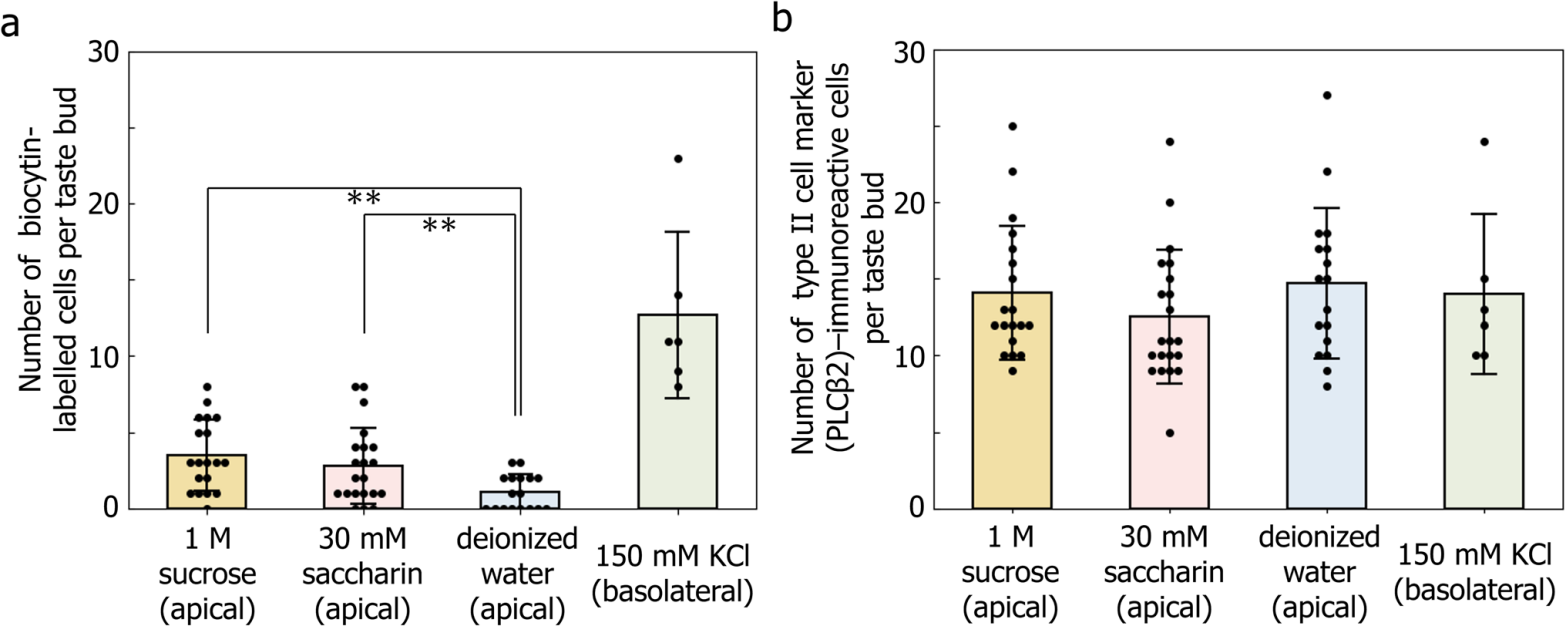
The number of biocytin-labeled cells per taste bud increased in response to sweet substances. **a** The number of biocytin-labeled cells per taste bud under each condition. Exposure to sucrose or saccharin significantly increased the number of biocytin-labeled cells compared with deionized water (**p < 0.01 unpaired *t* test with unequal variances). **b** The number of PLCβ2-immunoreactive cells per taste bud under each condition. No significant differences were observed among the groups (*p* = 0.51, one-way ANOVA). Error bars indicate SD

In peeled lingual epithelium preparations, fungiform taste buds typically exhibit an onion-shaped morphology (Fig. 1). Type II and III cells (PLCβ2- and SNAP-25-immunoreactive, respectively) extend apical processes toward the taste pore. Using confocal microscopy, optical sections were acquired through each taste bud, in which taste cell somata appeared as ring-like immunoreactive profiles. In this study, we used 1 M sucrose, a natural sweetener, or 30 mM saccharin, an artificial sweetener, to induce biocytin uptake in TBCs (Figs. 2 and 3a). In response to sucrose, the number of biocytin-labeled TBCs was 3.5 ± 2.3 (mean ± SD,* n* = 19) per taste bud, a significantly greater number of labeled TBCs than was observed with the control deionized water stimuli (1.1 ± 1.1, *n* = 17, *p* = 4.5 × 10^−4^, unpaired *t* test). Similarly, stimulation with 30 mM saccharin resulted in 2.8 ± 2.5 (*n* = 21) labeled cells, which was also significantly higher compared to the control (*p* = 7.8 × 10^−3^, unpaired *t* test). Because the number of TBCs per taste bud depends on the size of the taste bud, we compared the number of type II cells (PLCβ2-immunoreactive cells) per taste bud in each condition (Fig. 3b). No significant differences were observed among the groups (*p* = 0.51, one-way analysis of variance, ANOVA), indicating that biocytin uptake was induced by sweet stimulation rather than by variations in cell numbers and that these taste substances did not affect the number of PLCβ2-immunoreactive cells during stimulation.

After the uptake experiments, immunohistochemistry was performed to identify the cell types that took up biocytin in response to sweet stimuli. Among the biocytin-labeled TBCs, 74% (49/66 cells) of cells that responded to sucrose and 68% (40/59 cells) of cells that responded to saccharin were immunoreactive for PLCβ2. None of the biocytin-labeled TBCs were immunoreactive for SNAP-25. The remaining biocytin-labeled TBCs were non-immunoreactive for both PLCβ2 and SNAP-25, suggesting that they were either type I or immature type II cells. The results indicated that biocytin uptake induced by sweet stimuli occurs primarily in type II cells. To identify biocytin-labeled TBCs that are non-immunoreactive for type II and III markers, further experiments combining antibodies against type I markers (glutamate–aspartate transporter or nucleoside triphosphate diphosphohydrolase 2) with single-cell RT-PCR are warranted.

**Fig. 4:**
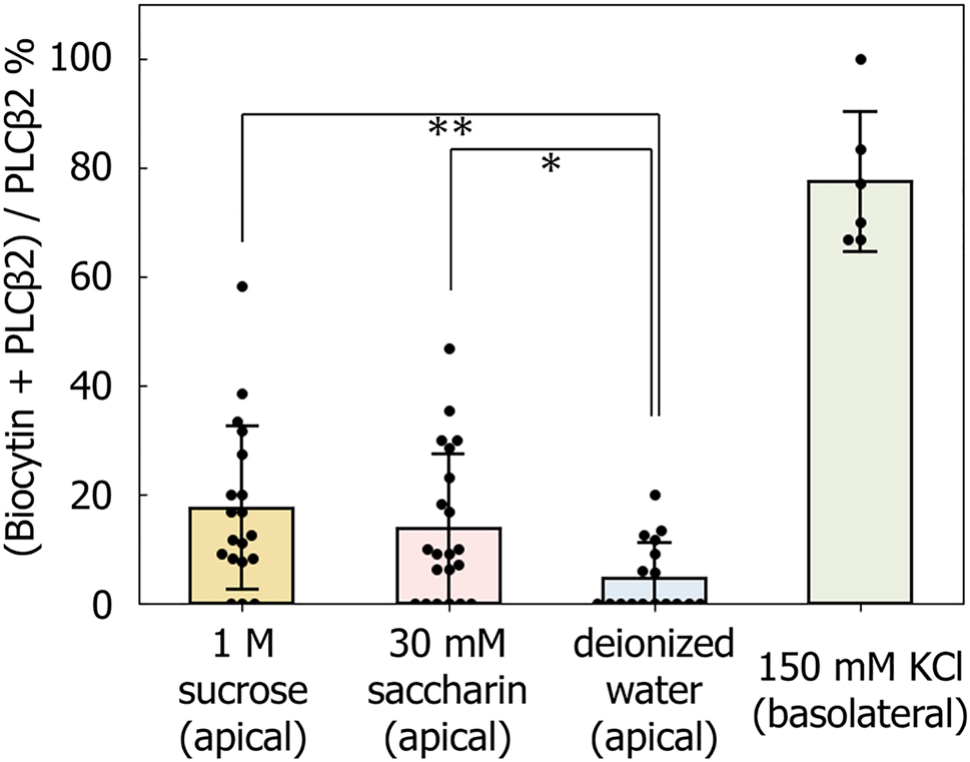
Biocytin uptake occurs in PLCβ2-immunoreactive cells after sweet stimuli. The ratio of biocytin-labeled PLCβ2-immunoreactive cells to the total PLCβ2- immunoreactive cells per taste bud under each stimulus is shown. Nearly all type II cells were labeled with biocytin following basolateral membrane exposure to high-K solution. Sucrose (1 M) and saccharin (30 mM) application to the apical membrane induced biocytin uptake in type II cells. The proportion of biocytin-labeled type II cells per taste bud in response to both natural and artificial sweet stimuli was significantly higher than that in response to deionized water (**p < 0.01, *p < 0.05, unpaired *t* test with unequal variances)

The proportion of biocytin-labeled type II cells per taste bud in response to stimulation with 1 M sucrose (17.4% ± 14.9%, *n* = 19) was significantly higher than that in response to deionized water (4.6% ± 6.4%, *n* = 17, *p* = 2.2 × 10^−3^, unpaired *t* test, Fig. 4). Likewise, 30 mM saccharin induced a significantly higher proportion of biocytin-labeled type II cells compared to the control (13.6% ± 13.7%, *n* = 21, *p* = 0.012, unpaired *t* test). These results suggest that, on average, approximately 11% of type II cells per taste bud respond to sweet stimuli, although considerable variability was observed among individual taste buds. This percentage represents the average net increase in cells responsive to sweet stimuli, calculated after subtracting the baseline response observed with the deionized water control.

Sweet substances can also be detected through a T1R2/T1R3-independent pathway involving glucose transporters and sodium-driven glucose symporters (Damak et al. 2003; Yasumatsu et al. 2020). Membrane potential changes in TBCs are likely to play pivotal roles in conveying sweet taste information to gustatory nerves through receptor-dependent and receptor-independent mechanisms [for a review, see von Molitor et al. (2021)]. Consequently, approximately 11% of type II cells, on average, may exhibit responses mediated by both pathways.

A previous single-cell RT-PCR study reported that approximately 50% of PLCβ2-GFP-expressing cells in the posterior taste buds expressed T1R2 and T1R3 mRNAs (Tizzano et al. 2008). Although our results suggest a lower proportion of sweet-responsive cells among type II cells, direct comparison is difficult because of the differences in taste bud location, experimental techniques, and evaluation criteria (taste bud-level vs. cell-level analyses) between the previous study and our own.

The marked variability in the proportion of sweet-responsive type II cells among fungiform taste buds suggests differences in their composition of sweet-, bitter-, and umami-sensitive cells. This supports the view that taste buds are functionally heterogeneous, with their tuning profiles shaped by their type II cell composition. Such heterogeneity may enhance broader taste discrimination by enabling diverse peripheral coding strategies.

Furthermore, the dynamic nature of TBCs likely contributes to the observed variability. Taste receptor cells have a short lifespan of approximately 10 days and are constantly replaced (Hamamichi et al. 2006; Perea-Martinez et al. 2013). If this renewal process occurs asynchronously and independently across taste buds, a heterogeneous distribution of sweet-responsive cells would inevitably occur at any given time point. Thus, the observed significant variability can be interpreted as a direct reflection of this asynchronous renewal cycle. This mechanism is considered to confer a crucial physiological advantage: it maintains consistent sweet taste perception at the system level by buffering against disruptions caused by cellular turnover.

## Conclusions

In this study, we developed a novel method to evaluate the number of TBCs that respond to sweet substances based on biocytin uptake. Using this approach, we demonstrated that biocytin labeling occurs primarily in type II cells in response to both natural and artificial sweet substances. Quantitatively, approximately 11% of type II cells per taste bud responded to sweet stimuli. Importantly, substantial variability in the number of sweet-responsive cells was observed across individual taste buds.

This variability suggests functional heterogeneity among fungiform taste buds, reflecting the observed differences in the proportion of sweet-sensitive type II cells. Such heterogeneity may indicate the divergent tuning properties of individual taste buds and contribute to consistent taste perception at the system level. Given the continuous turnover and short lifespan of TBCs, the asynchronous renewal of sweet-responsive cells across taste buds may act as a buffering mechanism, preventing the transient loss of sensitivity and ensuring the reliable detection of sweet stimuli over time.

## Data Availability

The data that support the findings of this study are available within the article; additional underlying data are available from the corresponding author on reasonable request.

## References

[CR1] Adler E, Hoon MA, Mueller KL, Chandrashekar J, Ryba NJ, Zuker CS (2000) A novel family of mammalian taste receptors. Cell 100(6):693–702 10761934 10.1016/s0092-8674(00)80705-9

[CR2] Chaudhari N, Roper SD (2010) The cell biology of taste. J Cell Biol 190(3):285–296. 10.1083/jcb.201003144 20696704 10.1083/jcb.201003144 PMC2922655

[CR3] Damak S, Rong M, Yasumatsu K, Kokrashvili Z, Varadarajan V, Zou S, Jiang P, Ninomiya Y, Margolskee RF (2003) Detection of sweet and umami taste in the absence of taste receptor T1r3. Science 301(5634):850–853. 10.1126/science.1087155 12869700 10.1126/science.1087155

[CR4] Finger TE, Danilova V, Barrows J, Bartel DL, Vigers AJ, Stone L, Hellekant G, Kinnamon SC (2005) ATP signaling is crucial for communication from taste buds to gustatory nerves. Science 310(5753):1495–1499. 10.1126/science.1118435 16322458 10.1126/science.1118435

[CR5] Hamamichi R, Asano-Miyoshi M, Emori Y (2006) Taste bud contains both short-lived and long-lived cell populations. Neuroscience 141(4):2129–2138. 10.1016/j.neuroscience.2006.05.061 16843606 10.1016/j.neuroscience.2006.05.061

[CR6] Iwamoto M, Takashima M, Ohtubo Y (2020) A subset of taste receptor cells express biocytin-permeable channels activated by reducing extracellular Ca(2+) concentration. Eur J Neurosci 51(7):1605–1623. 10.1111/ejn.14672 31912931 10.1111/ejn.14672

[CR7] Kashio M, Wei-Qi G, Ohsaki Y, Kido MA, Taruno A (2019) CALHM1/CALHM3 channel is intrinsically sorted to the basolateral membrane of epithelial cells including taste cells. Sci Rep 9(1):2681. 10.1038/s41598-019-39593-5 30804437 10.1038/s41598-019-39593-5 PMC6390109

[CR8] Mori Y, Eguchi K, Yoshii K, Ohtubo Y (2016) Selective expression of muscarinic acetylcholine receptor subtype M3 by mouse type III taste bud cells. Pflugers Arch 468(11–12):2053–2059. 10.1007/s00424-016-1879-5 27628900 10.1007/s00424-016-1879-5 PMC5138268

[CR9] Murray RG (1973) The ultrastructure of taste buds. In: Friedmann I (ed) The ultrastructure of sensory organs. North-Holland Publishing, Amsterdam, pp 1–81

[CR10] Nakao Y, Tateno K, Ohtubo Y (2022) Taste receptor cells generate oscillating receptor potentials by activating G protein-coupled taste receptors. Front Physiol 13:883372. 10.3389/fphys.2022.883372 35694396 10.3389/fphys.2022.883372 PMC9174655

[CR11] Nelson G, Hoon MA, Chandrashekar J, Zhang Y, Ryba NJ, Zuker CS (2001) Mammalian sweet taste receptors. Cell 106(3):381–390 11509186 10.1016/s0092-8674(01)00451-2

[CR12] Nelson G, Chandrashekar J, Hoon MA, Feng L, Zhao G, Ryba NJ, Zuker CS (2002) An amino-acid taste receptor. Nature 416(6877):199–202. 10.1038/nature726 11894099 10.1038/nature726

[CR13] Ogata T, Ohtubo Y (2020) Quantitative analysis of taste bud cell numbers in the circumvallate and foliate taste buds of mice. Chem Senses 45(4):261–273. 10.1093/chemse/bjaa017 32157267 10.1093/chemse/bjaa017

[CR14] Ohtubo Y (2021) Slow recovery from the inactivation of voltage-gated sodium channel Nav1.3 in mouse taste receptor cells. Pflugers Arch 473(6):953–968. 10.1007/s00424-021-02563-w 33881614 10.1007/s00424-021-02563-w

[CR15] Ohtubo Y, Yoshii K (2011) Quantitative analysis of taste bud cell numbers in fungiform and soft palate taste buds of mice. Brain Res 1367:13–21. 10.1016/j.brainres.2010.10.060 20971092 10.1016/j.brainres.2010.10.060

[CR16] Ohtubo Y, Suemitsu T, Shiobara S, Matsumoto T, Kumazawa T, Yoshii K (2001) Optical recordings of taste responses from fungiform papillae of mouse in situ. J Physiol 530(Pt 2):287–293. 10.1111/j.1469-7793.2001.0287l.x 11208976 10.1111/j.1469-7793.2001.0287l.x PMC2278412

[CR17] Perea-Martinez I, Nagai T, Chaudhari N (2013) Functional cell types in taste buds have distinct longevities. PLoS ONE 8(1):e53399. 10.1371/journal.pone.0053399 23320081 10.1371/journal.pone.0053399 PMC3540047

[CR18] Roper SD (2013) Taste buds as peripheral chemosensory processors. Semin Cell Dev Biol 24(1):71–79. 10.1016/j.semcdb.2012.12.002 23261954 10.1016/j.semcdb.2012.12.002 PMC3690797

[CR19] Takeuchi K, Seto Y, Ohtubo Y, Yoshii K (2011) Dye-permeable, voltage-gated channel on mouse fungiform taste bud cells. Brain Res 1373:17–24. 10.1016/j.brainres.2010.12.019 21167135 10.1016/j.brainres.2010.12.019

[CR20] Takeuchi K, Yoshii K, Ohtubo Y (2021) Age-related electrophysiological changes in mouse taste receptor cells. Exp Physiol 106(2):519–531. 10.1113/EP089104 33174320 10.1113/EP089104

[CR21] Taruno A, Vingtdeux V, Ohmoto M, Ma Z, Dvoryanchikov G, Li A, Adrien L, Zhao H, Leung S, Abernethy M, Koppel J, Davies P, Civan MM, Chaudhari N, Matsumoto I, Hellekant G, Tordoff MG, Marambaud P, Foskett JK (2013) CALHM1 ion channel mediates purinergic neurotransmission of sweet, bitter and umami tastes. Nature 495(7440):223–226. 10.1038/nature11906 23467090 10.1038/nature11906 PMC3600154

[CR22] Tizzano M, Dvoryanchikov G, Barrows JK, Kim S, Chaudhari N, Finger TE (2008) Expression of Galpha14 in sweet-transducing taste cells of the posterior tongue. BMC Neurosci 9:110. 10.1186/1471-2202-9-110 19014514 10.1186/1471-2202-9-110 PMC2596171

[CR23] von Molitor E, Riedel K, Krohn M, Hafner M, Rudolf R, Cesetti T (2021) Sweet taste is complex: signaling cascades and circuits involved in sweet sensation. Front Hum Neurosci 15:667709. 10.3389/fnhum.2021.667709 34239428 10.3389/fnhum.2021.667709 PMC8258107

[CR24] Yasumatsu K, Ohkuri T, Yoshida R, Iwata S, Margolskee RF, Ninomiya Y (2020) Sodium-glucose cotransporter 1 as a sugar taste sensor in mouse tongue. Acta Physiol (Oxf) 230(4):e13529. 10.1111/apha.13529 32599649 10.1111/apha.13529 PMC8284877

